# Highly Sensitive Microwave Sensors Based on Open Complementary Square Split-Ring Resonator for Sensing Liquid Materials

**DOI:** 10.3390/s24061840

**Published:** 2024-03-13

**Authors:** Chandu Ds, K. B. S. Sri Nagini, Rusan Kumar Barik, Slawomir Koziel

**Affiliations:** 1 School of Electronics Engineering, VIT-AP University, Amaravati 522237, India; srinagini.21phd7012@vitap.ac.in; 2 Engineering Optimization and Modeling Center, Reykjavik University, 102 Reykjavik, Iceland; rusanb@ru.is; 3 Faculty of Electronics, Telecommunications and Informatics, Gdańsk University of Technology, 80-233 Gdańsk, Poland

**Keywords:** distilled water, ethanol, methanol, microwave sensor, open complementary split-ring resonator, sensitivity

## Abstract

This paper presents high-sensitivity sensors based on an open complementary square split-ring resonator and a modified open complementary split-ring resonator operating at 4.5 GHz and 3.4 GHz, respectively. The sensors are designed for the detection of multiple liquid materials, including distilled water, methanol, and ethanol. The liquid under test is filled in a glass container loaded using a pipette. Compared to the conventional OCSSRR, the modified OCSSRR with multiple rings exhibits a higher frequency shift of 1200 MHz, 1270 MHz, and 1520 MHz for ethanol, methanol, and distilled water, respectively. The modified sensor also demonstrates a high sensitivity of 308 MHz/RIU for ethanol concentration which is the highest among the existing microwave sensors. The sensors in this manuscript are suitable for multiple liquid-material-sensing applications.

## 1. Introduction

Microwave resonator sensors have gained significant popularity in recent years due to their applications in addressing various technological challenges, particularly in the detection and analysis of liquid materials being tested. The presence of a material with specific dielectric properties or changes in the physical properties of the medium such as permittivity and loss tangent can modify the electromagnetic characteristics of the resonator element in the sensor. This modification leads to measurable changes in the resonant frequency. The transmission characteristics of the dielectric substrate are influenced by these properties, subsequently impacting the propagation of electromagnetic waves through the material. Microwave sensors are employed for material characterization in diverse fields including agriculture [1], medical care [2,3], and industrial applications [4].

Numerous microwave sensors have been developed to characterize various materials, including oils [5,6,7], coal [8,9], glucose [10,11,12], solids [13,14,15], gases [16,17,18], and gesomin [19]. In particular, microwave sensors are also widely used for detecting liquid samples (such as ethanol) based on the glass tube method [20,21], LC method [22], or microfluidic channel method [23,24]. From the results in the literature, it is found that the microfluidic channel method leads to the highest sensitivity of up to 268 MHz/RIU compared to other methods. Researchers have also implemented sensing of two liquid samples such as methanol/ethanol [25,26,27], water/ethanol [28,29,30], etc. Irrespective of the liquid fitting method, it is worth mentioning that the sensitivity of the sensor also depends on the number of rings in the split-ring resonator structure [29]. In a similar way, single-sensor structures capable of sensing multiple liquid samples such as ethanol/methanol/water have also been proposed [31,32,33]. The main sensing element of these microwave sensors are resonator-based as it offers several advantages like simple design, high performance characteristics, low cost, and ease of fabrication. For example, the split-ring resonator [26], complementary split-ring resonators (CSRR) [21], substrate-integrated waveguide resonator [25], Minkowski-like fractal resonator [31], complementary circular spiral resonator [28], gap waveguide cavity resonator [27], multiple split-ring resonator [33] and, multiple complementary split-ring resonator [29], which are coupled to a transmission line, have gained considerable attention for potential sensing applications with various liquid samples. Microwave sensors based on conventional complementary split-ring resonators have achieved a sensitivity of 268 MHz/RIU [24]. Another method to enhance the sensitivity involves the utilization of multiple complementary split-ring resonators by employing a microstrip line that results in a sensitivity of 269 MHz/RIU [29]. However, achieving high sensitivity with a maximum frequency shift for liquid material detection still remains a challenging task.

In this paper, an open complementary square split-ring resonator (OCSSRR) and a modified OCSSRR are proposed for sensing multiple liquid materials including distilled water, methanol, and ethanol. To load the test samples and detect the liquid material being tested, sensors typically require the use of an extra capillary tube, a microfluidic channel, or glass tubes/containers. In this study, a simple design and non-contact method is proposed for liquid detection utilizing a small glass container located perpendicular to the ground plane. The modified OCSSRR sensor demonstrates a high sensitivity of 308 MHz/RIU. These sensors exhibited high sensitivity, particularly for ethanol concentrations. The proposed sensors are validated through both numerical simulations and experimental testing.

## 2. Materials and Methods

The proposed sensors consist of three layers: the top conducting plane, dielectric layer, and bottom ground plane. The dielectric material used in this study is FR4 with a relative permittivity ($\varepsilon_{r}$) of 4.4, dielectric loss tangent (tanδ) of 0.02, and thickness of 1 mm. The top and bottom conducting layers are copper, with a thickness of 0.035 mm. Various standard solvents like distilled water (H_2_O), ethanol (C_2_H_5_OH), and methanol (CH_3_OH) are tested to assess the reliability and validate the efficiency of the sensor. The dielectric constants of these samples are 80.1, 24.5, and 32.7, respectively, and the corresponding dielectric loss tangents are 0.94, 0.65, and 0.12. A glass container is used for sensing the liquid. The inner and outer radius of the glass container are optimized to 6 mm and 7 mm, respectively, and the height of the container is 8 mm. The glass container is incorporated into the OCSSRR sensor to characterize the properties of liquid samples and the container is positioned perpendicular to the detection zone. The concentration of the liquid sample in the glass container is maintained at a pure 100% solution, with a volume of 10 μL dispensed incrementally.

**Figure 1:**
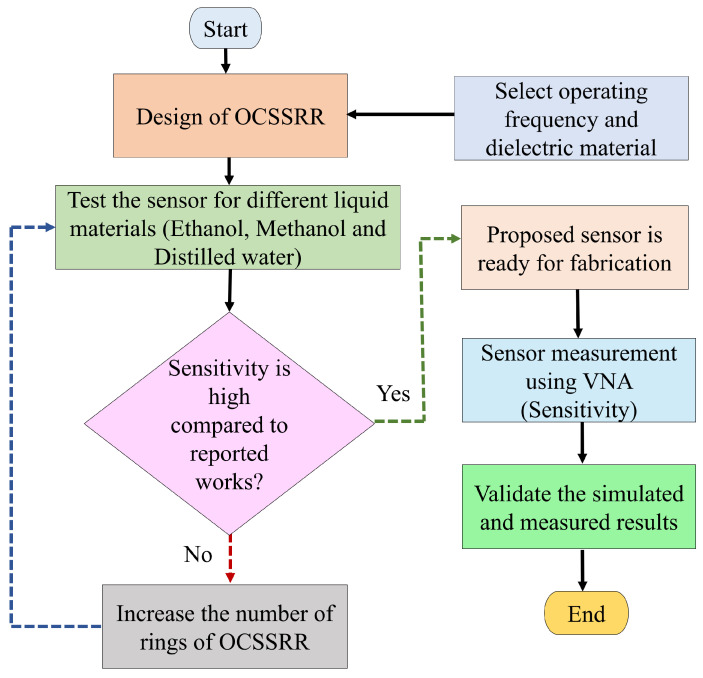
Design flow and methodology for sensing of liquid materials.

The methodology of the proposed sensor is illustrated in Figure 1. The detailed procedure for the design, analysis, and fabrication is outlined as follows:

1. Initially, a microwave sensor is designed by selecting the operating frequency and dielectric material.
2. The designed sensor is tested with different liquid materials such as ethanol, methanol, and distilled water.
3. In the next step, the sensitivity is calculated, and the sensor is tested to achieve the highest frequency shift.
4. If the proposed sensor fails to exhibit satisfactory sensitivity of at least 200 MHz/RIU, then the number of rings in the OCSSRR is increased to achieve the highest sensitivity.
5. The fabricated sensors are measured and validated using a Vector Network Analyzer (VNA) to determine the sensitivity.
6. Finally, the simulated and measured results are validated, and the sensor is ready for practical applications.

## 3. Sensors Design and Analysis

In this work, two liquid sensors, namely OCSSRR (or sensor 1) and modified OCSSRR (or sensor 2), are designed, and the structures are optimized to enhance the sensing performance. A comparison analysis is performed to determine the optimal performance of the most effective sensor.

### 3.1. Open Complementary Square Split-Ring Resonator (OCSSRR)—Sensor 1

**Figure 2:**
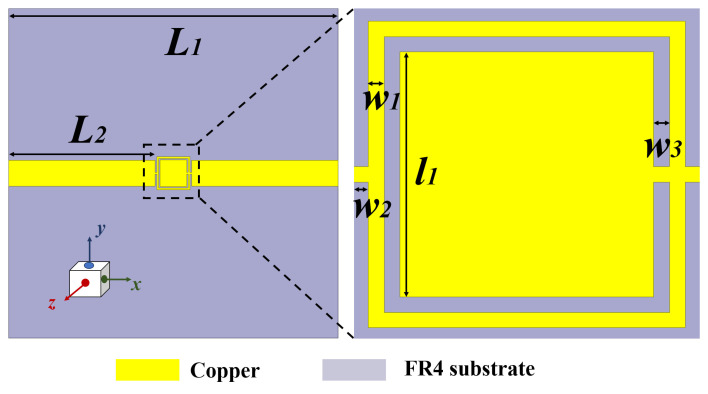
Top view of the OCSSRR sensor, and the optimized parameters are *L*_1_ = 40 mm, *L*_2_ = 17.8 mm, *l*_1_ = 3.2 mm, *w*_1_ = *w*_2_ = *w*_3_ = 0.2 mm.

The geometrical view of the OCSSRR (aslo called sensor 1) is shown in Figure 2 along with a zoomed-in view of the resonator and the optimized dimensions. The sensor consists of an OCSSRR and a microstrip line. The design evolution of the sensor can be explained as follows:

1. Initially, a single square patch is chosen (called structure 1) that resonates at a frequency of 6.5 GHz as shown in Figure 2. As there is no sensing element, structure 1 cannot function as a sensor.
2. A complimentary ring is added in structure 2, and the sensing region is positioned at the top plane. This is referred to as sensor 1.

The length of the sensor is *L*_1_ = 40 mm, the length of the microstrip line is *L*_2_ = 17.8 mm, the length of the sensing element is (*L*_1_ − 2 × *L*_2_) 4.4 mm, the length of the inner square ring is *l*_1_ = 3.2 mm, and the width of the complementary split-ring is *w*_3_ = 0.2 mm.

The optimized parameters are selected to achieve an operating frequency of 4.507 GHz for sensor 1. This is achieved by using the the empirical expression in Equation (1) as follows:

(1) $$f_{r}=\frac{1}{2\pi \sqrt{LC}}=\frac{c}{12\left[\left(l_{1}+w_{1}+w_{2}+w_{3}\right)\right]\sqrt{\frac{\varepsilon_{reff}+1}{2}}}$$

**Figure 3:**
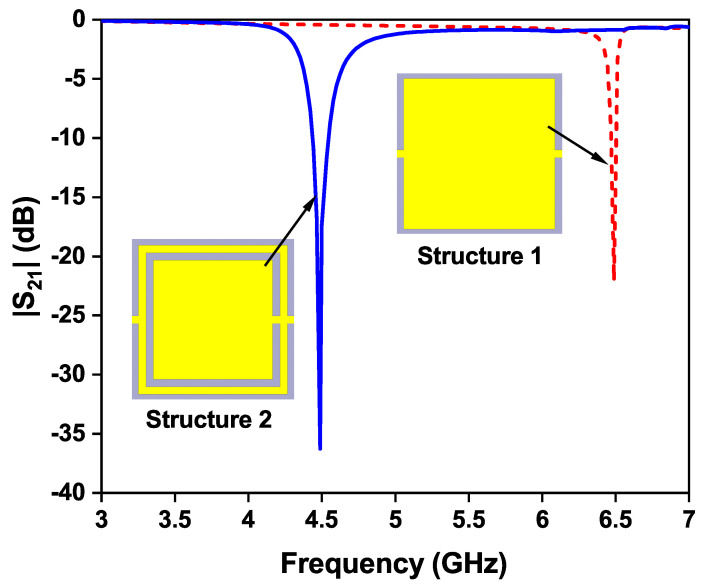
Transmission coefficient of structure 1 and structure 2.

The length of the split-ring resonator structure is carefully chosen to achieve the most effective frequency shift while minimizing changes in the material parameters of an unknown liquid solution. This selection aims to optimize the sensor’s performance in detecting and responding to variations in the liquid solution. Due to the addition of the complementary ring, the resonant frequency of structure 2 is shifted towards higher wavelengths, and sensor 1 operates at a frequency of 4.507 GHz, as shown in Figure 3, without loading the glass container.

**Figure 4:**
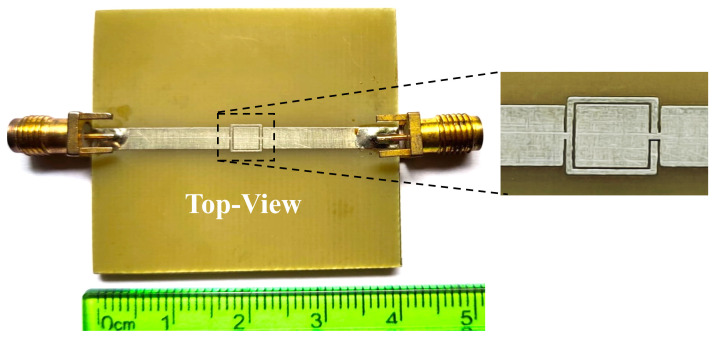
Photograph of the fabricated OCSSRR sensor.

**Figure 5:**
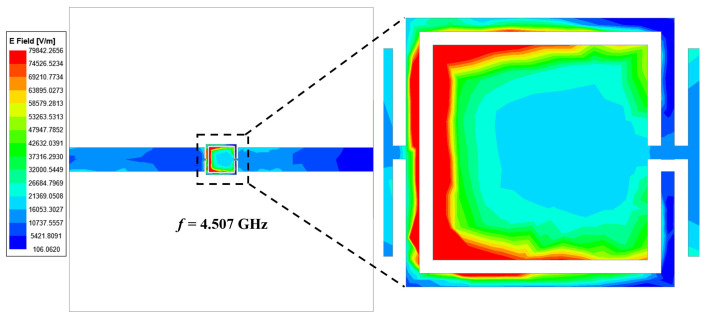
Simulated electric field distribution of sensor 1.

A prototype of the fabricated model is shown in Figure 4. The symmetrical design of the OCSSRR geometric structure optimizes the coupling of electromagnetic fields, resulting in the enhanced transfer of input microwave power into the central detection region. This optimization maximizes the effectiveness of the sensor in detecting the liquid samples. This can be verified from the simulated electric field of the sensing region in the resonator without a sample, as depicted in Figure 5. The electric fields are obtained through comprehensive full-wave electromagnetic model simulations of the OCSSRR. It can be observed that the maximum field is distributed near the edges of the OCSSRR at the resonant frequency of 4.507 GHz. The concentration of energy will be maximum at the center of the sensor, and hence, the sensitivity can be maximized.

#### Parametric Analysis of OCSSRR—Sensor 1

**Figure 6:**
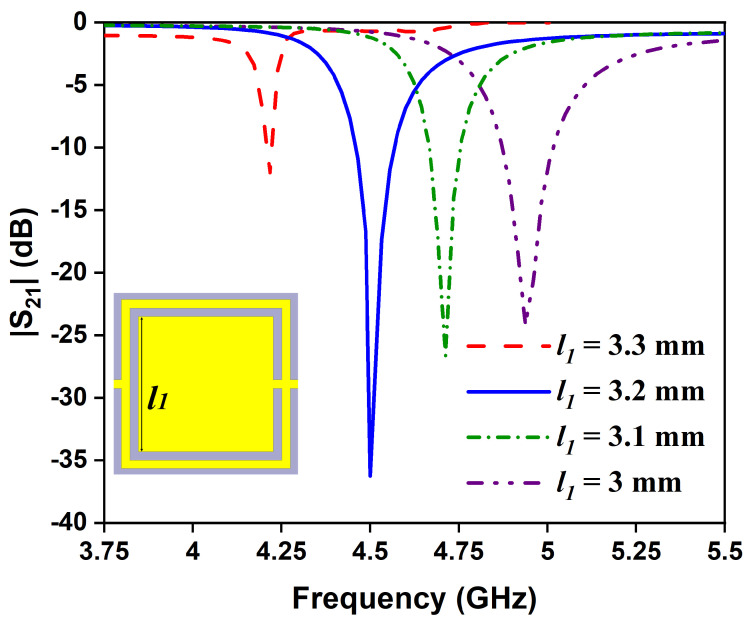
Effect of OCSSRR length on the resonant frequency.

**Figure 7:**
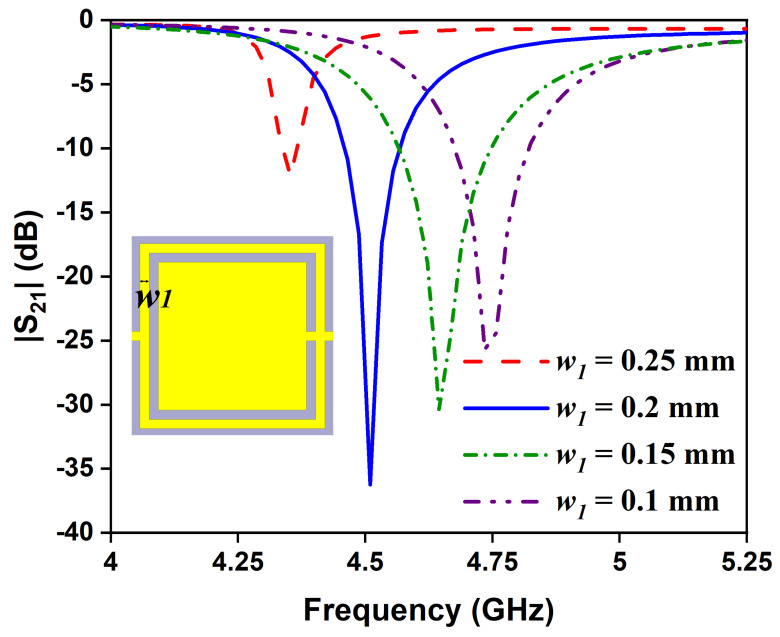
Effect of resonant frequency due to the variation of width.

The influence of geometrical parameters of the sensor are studied by varying the length of the complementary split-ring (*l*_1_) and the width of the split-ring (*w*_1_). Figure 6 shows the transmission coefficient of the sensor due to changes in the length of OCSSRR from 3 to 3.3 mm. As the length is inversely proportional to the resonant frequency, the region of operation shifts from 4.93 to 4.21 GHz with increasing length. For *l*_1_ < 3.2 mm and *l*_1_< 3.2 > 3.2 mm, the transmission coefficient is only around −12 dB and −25 dB, respectively. In order to achieve high sensitivity, the length of the OCSSRR is chosen as 3.2 mm which results in a perfect transmission zero. Similarly, as the width (*w*_1_) varies from 0.1 to 0.25 mm, the resonant frequency shifts from 4.74 to 4.35 GHz, as shown in Figure 7. The perfect transmission zero occurs when the width is 0.2 mm. Therefore, the optimized width of the OCSSRR is chosen as 0.2 mm to achieve improved performance characteristics of the sensor.

### 3.2. Modified Open Complementary Square Split-Ring Resonator (OCSSRR)—Sensor 2

**Figure 8:**
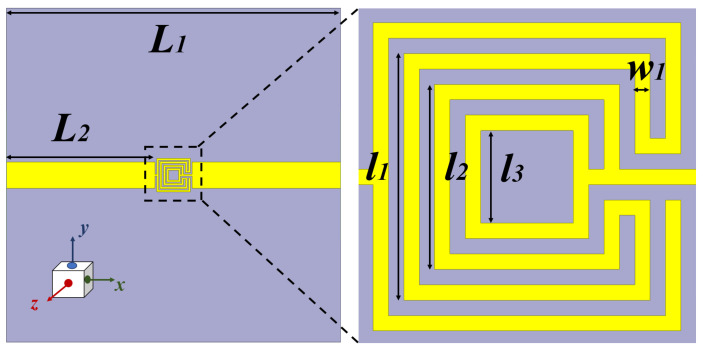
Geometry of the modified OCSSRR sensor.

**Figure 9:**
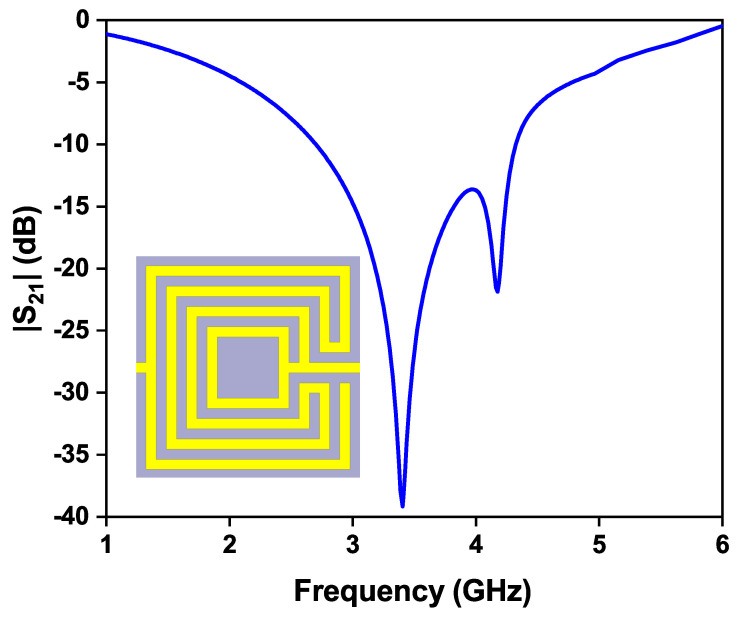
Transmission coefficient plot of sensor 2.

The modified OCSSRR is loaded on the top metallic plane fed with a microstrip transmission line shown in Figure 8. Sensor 2 consists of four open square-shaped split-ring resonators which are loaded on the center of the top metallic plane. The geometrical parameters of the modified sensor are as follows: the length of the sensor is *L*_1_ = 40 mm, the length of the microstrip line is *L*_2_ = 17.8 mm, the lengths of the square rings are *l*_1_ = 3.2 mm, *l*_2_ = 2.4 mm, and *l*_3_ = 1.2 mm, and the width of the complementary split-ring is *w*_1_ = 0.2 mm. By increasing the number of square slots, the resonant frequency further shifts to lower bands compared to the OCSSRR sensor. The modified OCSSRR (sensor 2) is designed by increasing the number of rings in sensor 1. Sensor 2 resonates at a frequency of 3.404 GHz with enhancement in bandwidth as illustrated in Figure 9. The empirical expression given in Equation (2) is used to derive the theoretical operating frequency from the dimensions of sensor 2.

(2) $$f_{r}=\frac{1}{2\pi \sqrt{LC}}=\frac{c}{8\left[\sum_{i=1}^{3}\left(l_{i}+w_{i}\right)\right]\sqrt{\frac{\varepsilon_{reff}+1}{2}}}$$

**Figure 10:**
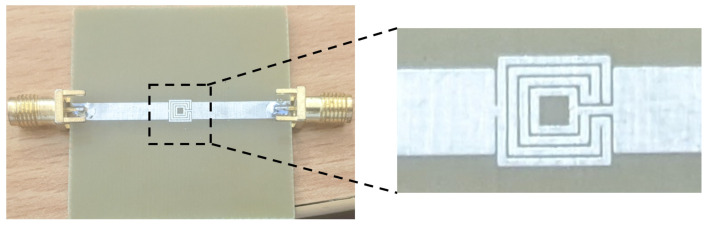
Fabricated prototype of the modified OCSSRR.

**Figure 11:**
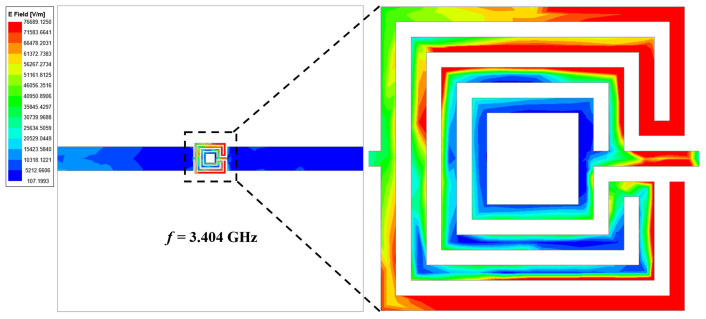
Electric fielddistribution of modified OCSSRR sensor.

The photograph of the fabricated prototype is shown in Figure 10, and the electric field distribution of the modified structure is illustrated in Figure 11. It can be observed that the maximum field is concentrated at the modified OCSSRR resonator which is located at the center of the structure. Consequently, it is advisable to position the glass container in this particular area.

### 3.3. Performance Analysis of Sensor 1 and Sensor 2

#### 3.3.1. Response of OCSSRR for Different Concentrations of Distilled Water

**Figure 12:**
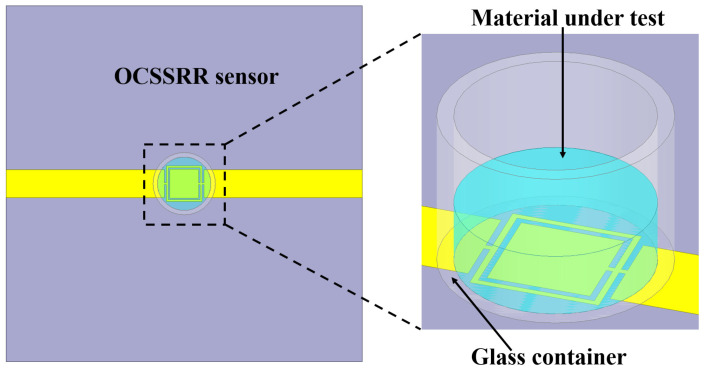
OCSSRR sensorwith liquid material under test.

The glass container is kept at the position at which the electric field intensity is maximum as shown in Figure 12. The variation in dielectric constants results in a frequency shift which can be observed as a result of employing the perturbation method. The optimum size of the detection region is selected to maximize the variation in stored electrical energy within ethanol/water droplets as their volumetric concentrations change. This choice aims to achieve a substantial shift in frequency, enhancing the sensitivity of the device for variation in concentrations. Distilled water samples with varying concentration are introduced into the glass container by using a pipette. Each liquid sample exhibits dielectric effects that interact with the electric fields within the sensor region, and these interactions are responsible for the shift in resonant frequency of the OCSSRR sensor.

**Figure 13:**
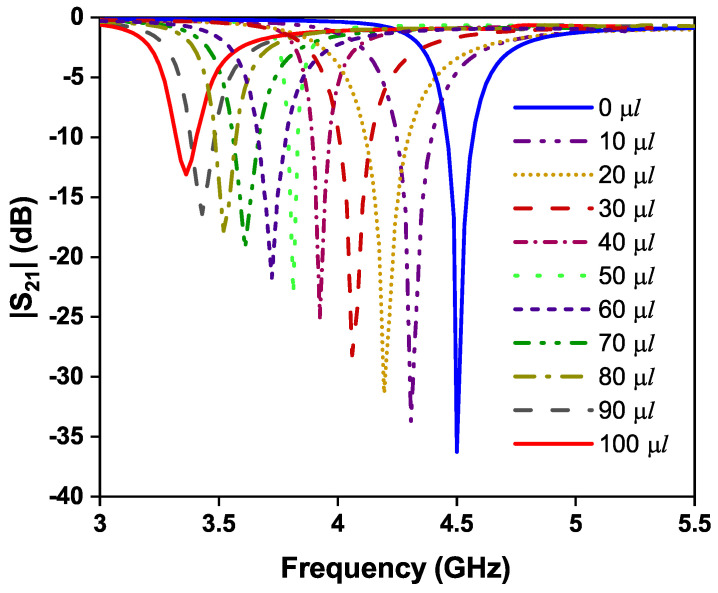
Variation inresonant frequency for different concentrations of distilled water.

**Figure 14:**
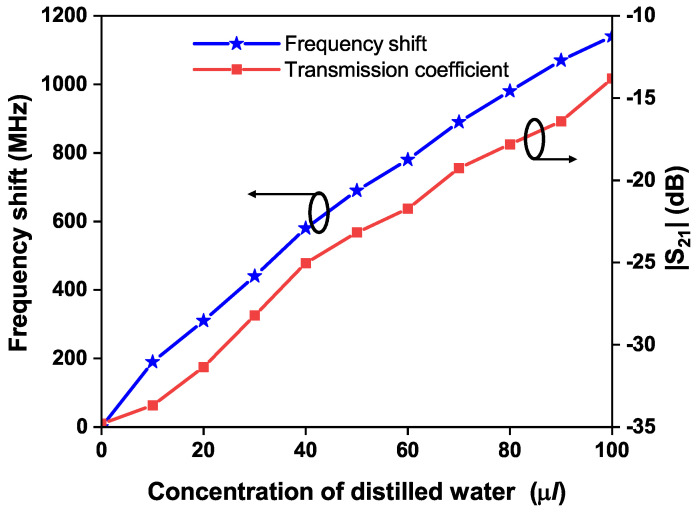
Frequency shift and transmission coefficient of sensor 1 for different concentrations of distilled water.

Figure 13 illustrates the transmission coefficients of the OCSSRR-based sensor for different fractions of distilled water. It becomes evident from Figure 14 that as the concentration of water varies from 10% to 100%, there is a gradual decrease in the resonant frequency from 4.31 to 3.36 GHz. The sensor operates at a resonant frequency of 4.507 GHz without the glass container, and by loading the glass container, there is an insignificant shift in the resonant frequency. Thus, the sensor with the presence of a container (air-filled) operates at a frequency of 4.5 GHz. The shift in resonant frequency is due to the change in the effective permittivity which is due to the added mass of the sensor. Table 1 provides the shift in the resonant frequency of the OCSSRR sensor for different concentrations of water levels; the maximum shift of 1140 MHz is obtained when the concentration of distilled water is 100 μL, and a minimum shift of 190 MHz is observed for a concentration of 10 μL.

When a liquid sample is introduced into an empty glass container, its permittivity becomes more above unity, resulting in an increase in the phase constant. When a liquid sample with a permittivity above unity is introduced into the glass container, it causes an increase in the phase constant (β) due to the change in the electromagnetic properties of the medium inside the container. This increase in “β” leads to a decrease in the resonant frequency required to satisfy the phase condition β × L = π/2. The amount of this shift in the resonance from its initial value (*f*_0_) depends on the liquid concentration present in the container. The primary factor behind this change is the high-loss liquid, which induces greater perturbation in the radiated near field compared to a lower-loss fluid, resulting in a lower resonant frequency.

#### 3.3.2. Modified OCSSRR for Sensing Different Concentrations of Distilled Water

**Figure 15:**
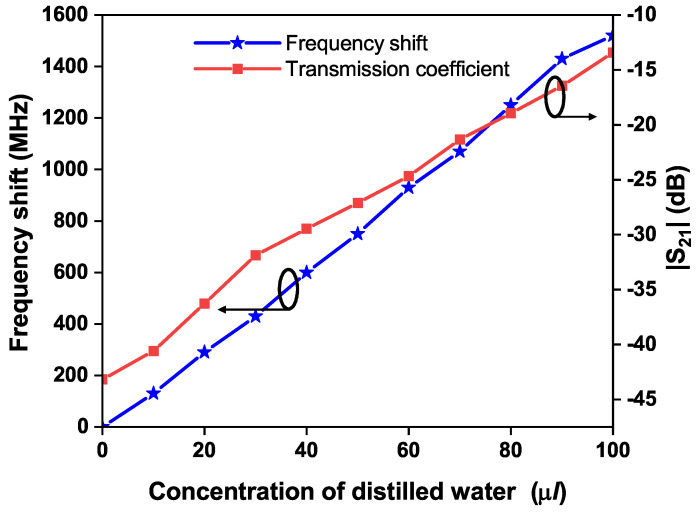
Frequency shift and transmission coefficient of sensor 2 for different concentrations of distilled water.

In the presence of a glass container filled with air, the modified OCSSRR resonates at a frequency of 3.4 GHz. Upon introducing liquid samples into the container, the electric fields near the resonator interact with the liquid material under test, so that the resonant frequency shifts slightly to lower frequencies. Table 1 provides the shift in resonant frequency for different concentrations of distilled water in steps of 10 μL loaded in the glass container. There is shift of 1520 MHz in the resonant frequency when the container is filled with 100 μL as shown in Figure 15. Compared to the OCSSRR sensor, the modified sensor provides a maximum shift of 1520 MHz in the resonant frequency, and this enhancement is achieved by opening the split ring with multiple rings, so that intensive electric fields are perturbed. The relative frequency shift is characterized as the ratio of the frequency change to that of the resonant frequency of the sensor under no-load conditions. The relative frequency shift Δ*f*_rel_ can be calculated by using Equation (3):

(3) $$\Delta f_{\mathrm{rel}}=\frac{\Delta f}{f_{r}}$$

where Δ*f* represents the change in frequency of the sensor, and *f*_r_ denotes the resonant frequency of the sensor under no-load conditions. Table 2 shows the relative frequency shift for different concentrations of distilled water for sensor 1 and sensor 2.

## 4. Results and Discussion

**Figure 16:**
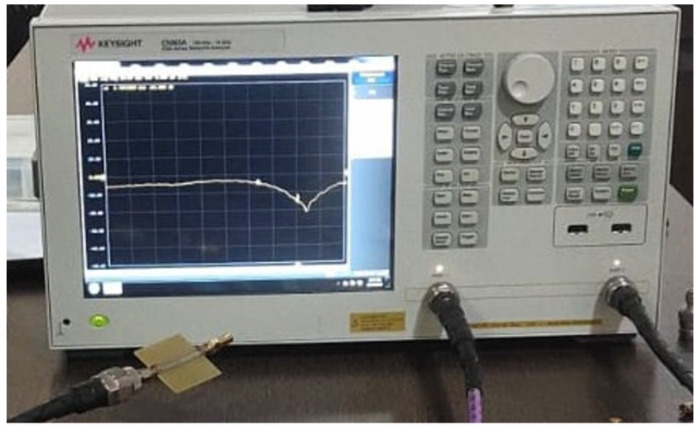
Photograph of the measurement of transmission coefficient using vector network analyzer.

**Figure 17:**
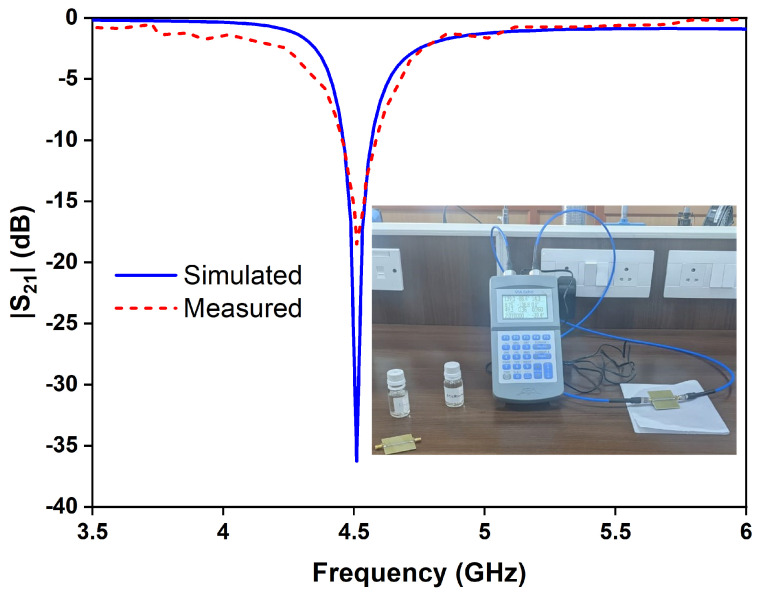
Simulated and measured results of transmission coefficient of the OCSSRR sensor with measurement setup in inset.

The transmission coefficients of the OCSSRR and modified OCSSRR sensors are measured using the Keysight E5063A vector network analyzer as shown in Figure 16. The simulated and measured values of transmission coefficient are plotted as shown in Figure 17. The measurement setup and the liquid samples being tested are shown in the inset.

### 4.1. Liquid Materials under Test

**Figure 18:**
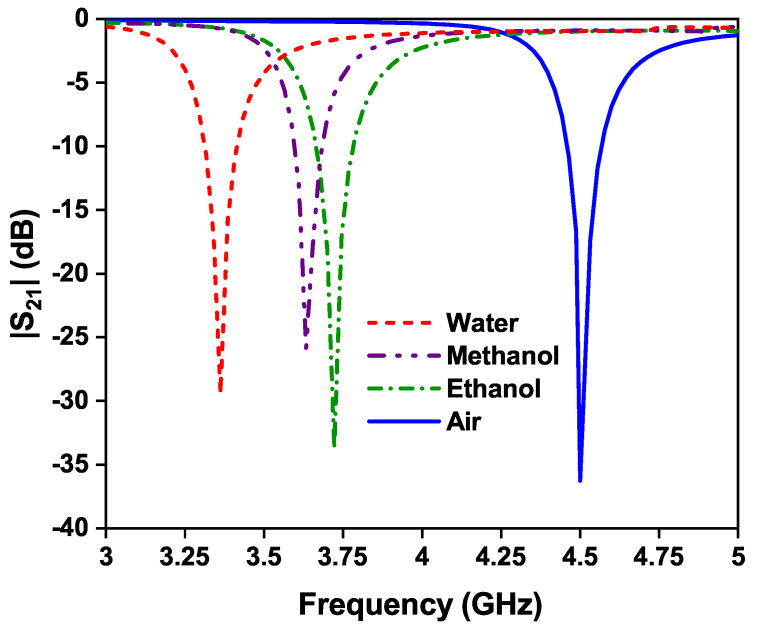
Effect of different liquid materials on the resonant frequency of the OCSSRR sensor.

The liquid sample detection was performed based on different liquid materials under test, by locating the sample at the center of the sensor, which is shown in Figure 18. As the test sample is loaded over the OCSSRR resonator, the resonant frequency will be changed. The change in resonant frequency will also depends on the sample concentration. The effect of the resonant frequency by testing different liquid materials is shown in Figure 18. A highest shift of 1.14 GHz is obtained, by loading distilled water in the glass container, since the relative permittivity of the material is inversely proportional to the frequency. There is a shift of 0.87 GHz and 0.78 GHz in the resonant frequency when methanol and ethanol liquid materials are loaded in the glass container, respectively.

**Figure 19:**
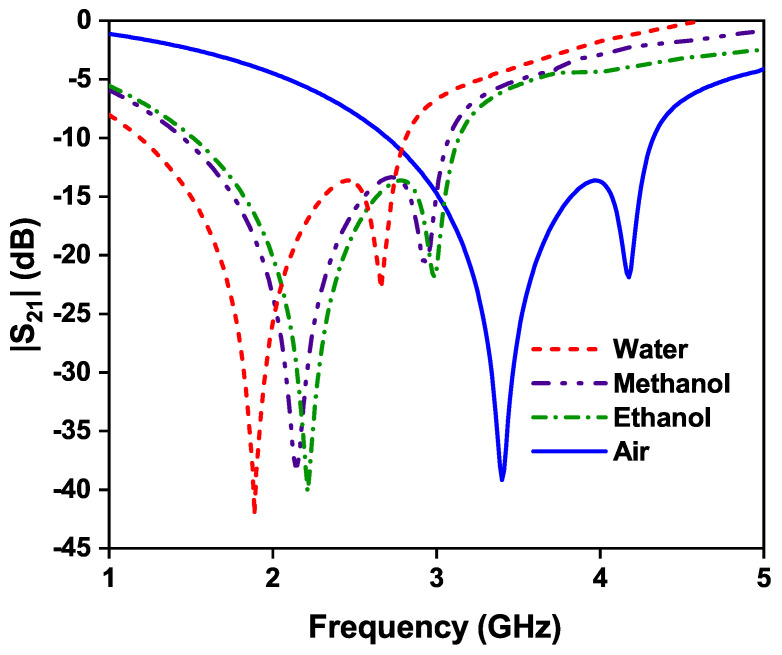
Effect of different liquid materials on the resonant frequency of modified OCSSRR sensor.

For the case of modified OCSSRR, the highest shift in the resonant frequency is 1.52 GHz for distilled water which has a high relative permittivity compared to methanol and ethanol. The shift in frequency for methanol and ethanol are 1.27 GHz and 1.2 GHz, respectively, as shown in Figure 19.

### 4.2. Sensitivity Analysis

The frequency response of the sensor depends on the relative permittivity of the different liquid material under test. The key parameter used to characterize the performance of the sensor is sensitivity. Sensitivity can be calculated by using Equation (4) [34],

(4) $$S=\frac{\Delta f}{\Delta n}\;\left(\mathrm{MHz}/\mathrm{RIU}\right)$$

where Δ*f* is the shift in resonant frequency, and Δn is the change in refractive index. The sensitivities of the different liquid materials are listed in Table 3. In the presence of a glass container filled with air, the modified OCSSRR resonates at a frequency of 3.4 GHz. For the case of the ethanol liquid, the resonant frequency is 2.2 GHz, and the shift in the resonant frequency is 1200 MHz. The change in the refractive index Δ*n* is 3.89 for the case of ethanol. Therefore, the measured value of sensitivity for ethanol is 308 MHz/RIU.

**Figure 20:**
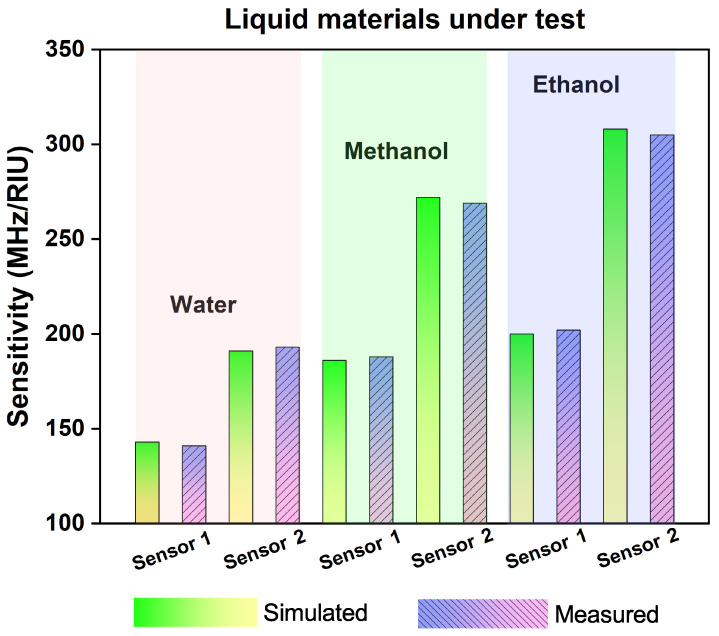
Simulated and measured sensitivity of OCSSRR and modified OCSSRR sensors.

This is the maximum sensitivity of the modified OCSSRR sensor. The OCSSRR with multiple rings demonstrates superior sensitivity compared to the single-ring OCSSRR for all of the liquid samples as shown in Figure 20. The comparison of performance characteristics of the proposed sensor with existing sensors is listed in Table 4. Sensor 2 proposed in this work, based on a Modified OCSSRR, demonstrates promising performance in sensing variations in the refractive index of distilled water, ethanol, and methanol samples. With a substantial frequency shift of 1200 MHz upon sample introduction, the sensor exhibits high sensitivity of 308 MHz/RIU compared to other existing sensors.

## 5. Conclusions

This paper proposes highly sensitive microwave sensors utilizing open complementary square split-ring resonators (OCSSRR). The single-ring OCSSRR operates at 4.5 GHz, and the modified OCSSRR with three additional rings has perfect transmission zero at 3.4 GHz, favorable for liquid material detection. Distilled water, methanol, and ethanol samples are placed in glass containers on top of the sensor’s detection zone. Interaction with the electric fields near the resonator induced significant changes in the transmission response, facilitating detection in both of the sensors. However, the modified OCSSRR sensor operating at 3.4 GHz exhibits remarkable sensitivities of 308 MHz/RIU, 272 MHz/RIU, and 191 MHz/RIU for ethanol, methanol, and distilled water, respectively, positioning it as a promising choice for liquid-material-detection applications.

## Figures and Tables

**Table 1:** Comparison of shift in resonant frequency of sensor 1 and sensor 2 for different concentrations of distilled water.

		OCSSRR	(Sensor 1)		
**CDW** **(μL)**	20	40	60	80	100
**RF** **(GHz)**	4.19	3.92	3.72	3.52	3.36
**S_21_** **(dB)**	−31.36	−25.03	−21.73	−17.82	−13.81
**FS** **(MHz)**	310	580	780	980	1140
		**Modified**	**OCSSRR**	**(Sensor 2)**	
**CDW** **(μL)**	20	40	60	80	100
**RF** **(GHz)**	3.11	2.8	2.47	2.15	1.88
**S_21_** **(dB)**	−36.26	−29.47	−24.66	−18.95	−13.45
**FS** **(MHz)**	290	600	930	1250	1520

CDW—concentration of distilled water; RF—resonant frequency; FS—frequency shift.

**Table 2:** Relative frequency shift for different concentrations of distilled water for sensor 1 and sensor 2.

		OCSSRR	(Sensor 1)		
**CDW** **(μL)**	20	40	60	80	100
**FS** **(MHz)**	310	580	780	980	1140
**RFS**	0.068	0.128	0.173	0.217	0.253
		**Modified**	**OCSSRR**	**(Sensor 2)**	
**CDW** **(μL)**	20	40	60	80	100
**FS** **(MHz)**	290	600	930	1250	1520
**RFS**	0.085	0.176	0.273	0.367	0.447

CDW—concentration of distilled water; RFS—resonant frequency shift; FS—frequency shift.

**Table 3:** Performance of OCSSRR and modified OCSSRR sensors for different liquid materials being tested.

		OCSSRR	(Sensor 1)		
**MUT**	**Frequency** **(GHz)**	**S_21_** **(dB)**	**Frequency** **Shift (GHz)**	**Relative** **Permittivity**	**Sensitivity** **(MHz/RIU)**
**Air** **(No Load)**	4.5	−36.26	-	-	-
**Ethanol**	3.72	−33.73	780	24	200
**Methanol**	3.63	−25.82	870	32	186
**Water**	3.36	−19.41	1140	80	143
		**Modified**	**OCSSRR**	**(Sensor 2)**	
**MUT**	**Frequency** **(GHz)**	**S_21_** **(dB)**	**Frequency** **Shift (GHz)**	**Relative** **Permittivity**	**Sensitivity** **(MHz/RIU)**
**Air** **(No Load)**	3.4	−39.16	-	-	-
**Ethanol**	2.2	−40.16	1200	24	308
**Methanol**	2.13	−37.92	1270	32	272
**Water**	1.88	−42.17	1520	80	191

**Table 4:** Performance comparison with existing sensors.

Ref.	Type of Resonator	Sample	RF (GHz)	FS (MHz)	Sensitivity (MHz/RIU)	LFM
[21]	CSRR	ethanol	2.96	110	23	glass tube
[20]	CSRR	ethanol	2.5	180	46	poly propylene tube
[31]	MLF	water/ ethanol/ methanol	1.97	50	52	glass capillary
[32]	CSRR	water/ ethanol/ methanol	6	200	77	PTFE tube
[30]	CSRR	water/ ethanol	5.37	970	125	microfluidic channel
[33]	MSRR	water/ ethanol/ methanol	2.1	420	128	capillary glass
[25]	SIW	ethanol/ methanol	4.4	600	153	glass capillary
[26]	SRR	ethanol/ methanol	2.6	600	153	plastic tube
[27]	GWCR	ethanol/ methanol	5.4	610	156	microfluidic channel
[22]	Series LC	ethanol	1.91	610	156	microfluidic channel
[28]	CCSR	water/ ethanol/ milk	2.4	800	205	precision pipette
[23]	CSRR	ethanol	4.72	550	212	microfluidic channel
[24]	CSRR	ethanol	2.226	1046	268	microfluidic channel
[29]	MCSRR	water/ ethanol	2.45	1050	269	capillary tube
TW	Modified OCSSRR	water/ ethanol/ methanol	3.4	1200	308	glass container

RF—resonant frequency; FS—frequency shift; LFM—liquid filling method; MSRR—multiple split-ring resonator; CCSR—complementary circular spiral resonator; CSRR—complementary split-ring resonator; SRR—split-ring resonator; GWCR—gap waveguide cavity resonator; SIW—substrate-integrated waveguide; MLF—Minkowski-like fractal; MCSRR—multiple complementary split-ring resonator; TW—this work.

## Data Availability

Data are contained within the article.
